# Spirituality in Australian Health Professional Practice: A Scoping Review and Qualitative Synthesis of Findings

**DOI:** 10.1007/s10943-023-01840-5

**Published:** 2023-06-12

**Authors:** Heather So, Lynette Mackenzie, Chris Chapparo, Judy Ranka, Mary Ann McColl

**Affiliations:** 1 Discipline of Occupational Therapy, School of Health Sciences, Faculty of Medicine and Health, University of Sydney, Camperdown, NSW 2006 Australia; 2 The Faculty of Health Sciences, Queen’s University, Kingston, ON Canada

**Keywords:** Australia, Health professional, Spirituality, Spiritual care, Religion

## Abstract

This study explores how spirituality is integrated into practice across the different Australian health professions. Utilising the Joanna Briggs Institute’s (JBI) protocol, six databases were searched, and sixty-seven articles were finally included. To present the findings, a qualitative synthesis was used. ‘Meaning’ and ‘purpose in life’ were found to be key to many spirituality definitions. The most frequently reported approach for Australian health professionals (HPs) in asking about client spirituality was using one or two questions within a comprehensive assessment. Major facilitators included a holistic care approach and prior training, whereas a key barrier was a lack of time.

**Supplementary Information:**

The online version contains supplementary material available at 10.1007/s10943-023-01840-5.

## Introduction

Spirituality encompasses important existential questions such as life meaning and purpose, the Transcendent (the spiritual world/the Divine) and connectedness (Puchalski et al., 2014), and yet it remains an often-neglected topic of conversation with Australian healthcare clients (Bloomer et al., 2022). This situation is compounded by the myriad of professional definitions and personal meanings around terms such as ‘spirituality’, ‘religion’ and ‘spiritual care’ (Murgia et al., 2020; Tavares et al., 2022). While reviews of spirituality definitions exist (de Brito Sena et al., 2021), the search for definitions which suit the Australian healthcare setting is problematic as the majority of literature regarding spirituality in healthcare originates from the United States (Rombola, 2019; Williams & Sternthal, 2007). The spiritual landscape of Australia is unique (Best et al., 2023). Australia combines a growing cultural and religious diversity (Australian Bureau of Statistics, 2017, 2022a), and unique indigenous outlook (Brodie et al., 2021), with a mainstream secular discomfort in discussing spirituality and religion (McCrindle, 2017).

In addressing client spirituality in healthcare, HPs represent the frontline as the first points of contact for clients. It has been stated that HPs should “recognise and respond to spiritual needs”; “provide spiritual support” and provide “referral as required” to spiritual experts (e.g. spiritual care practitioners and faith representative chaplains) (Holmes, 2018, p. 5). Yet, Australian healthcare presents a patchwork of approaches to client spirituality. The National Safety and Quality Health Service (NSQHS) Standards describes high-performing person-centred care as involving sensitivity to spiritual, cultural and religious needs and preferences (ACSQH, 2011, 2018). However, the majority of the Australian Health Practitioner Registration Agency (AHPRA) codes of conduct place a heavy emphasis on culture, and do not mention spirituality (AHPRA & National Boards, 2014, 2022). At a state/territory level, there are few spirituality resources and they are often directed primarily at those considered to be ‘spiritual experts’ (Queensland Government, 2020; Spiritual Health Association, 2020). Therefore, this study explores the literature of spiritual generalist Australian HPs to discover how they approach client spirituality.

## Background

A growing body of international evidence demonstrates the importance of spirituality to the health of clients, underlining the significance of appropriate screening or assessment (Koenig, 2015). By not addressing spiritual life, healthcare professionals may be ignoring, what some clients consider an important occupational area and source of coping through suffering (Eyres et al., 2018; Roze des Ordons et al., 2018). Australians have also reported that they would like their healthcare team to discuss spiritual beliefs more (Best et al., 2014, 2015; Hilbers et al., 2010). An Australian study found that clients wanted the whole health care team to be aware of spiritual beliefs which were important to them, and to see “spirituality as an integral aspect of care” (Gardner et al., 2020, p. 201).

Internationally, many spiritual assessments and screening tools are available across different disciplines, and reviews of these have been conducted (Gray, 2015; Hodge, 2005; Rumbold, 2007; Saguil & Phelps, 2012). However, it is not clear which methods of asking about spirituality are reported by Australian HPs, or what helps and hinders engaging with client spirituality. International research into the facilitators and barriers to addressing spirituality in healthcare has been growing (Best et al., 2016a; Koenig, 2015). While Australian studies exist for some individual professions (Best et al., 2016b; Smyth & Allen, 2011), this scoping review employs literature from many Australian health professions to create an overall map of facilitators and barriers to addressing spirituality.

A preliminary investigation of Medline, Embase and PsycInfo databases indicated that there are no existing scoping reviews on this topic. Therefore, the aim of this study was to methodically chart how spirituality is understood and addressed by Australian HPs in practice.

## Methods

A scoping review, using the JBI methodology, was selected in order to map a broad range of literature, as it allows for both surveying current knowledge and understanding the need for further research (Peters et al., 2020). Qualitative synthesis is an approach that has been used to present findings in recent scoping reviews (Bright et al., 2022; Wu et al., 2019). This study generated a conceptual model using thematic analysis (Braun & Clarke, 2021) to organise and present the study findings in relation to the review questions.

### Review Questions

This scoping review looked at the question: how do Australian HPs integrate spirituality into their practice? Specific review questions were:

1. How do Australian HPs define key spirituality concepts, including “spirituality”, “spiritual care” and “religion”?
2. How do Australian HPs ask about and respond to their clients’ spirituality?
3. What are the facilitators and barriers to addressing client spirituality?

### Search Strategy

With the aid of a health sciences librarian, a three-step approach was used in line with JBI recommended methodology (Peters et al., 2020). An initial search was conducted of six databases, followed by an analysis of the MeSH terms used in the literature. An example of the Medline search strategy has been appended (see Appendix I). The six databases searched included Medline, Embase, PsycInfo, Cinahl, Web of Science and Scopus, searched up until 24th June 2022.

### Inclusion Criteria

#### Participants

Australian HPs from any Australian state or territory who had clinical roles and were spiritual generalists were defined using two representative lists: the NSW Health Awards 2019 (NSW Government Health, 2021) and AHPRA registration standards (AHPRA & National Boards, 2019). These terms were then added to all related MeSH terms for health professional. Multidisciplinary studies were included only if the relevant HP data could be extracted discretely.

#### Concept

Concepts included “spiritual*”, to encompass “spiritual”, “spirituality” and “spiritual care”; and “religio*”, to encompass “religion” and “religiosity”.

#### Context

This scoping review focused on Australia, including all its states and territories. International studies that included Australian participants were included only if the Australian data could be extracted discretely.

#### Types of Sources

For this scoping review, the sources of information allowed for the inclusion of peer reviewed articles such as quantitative and qualitative studies, position papers, guidelines, and other summaries. Due to the wide range of terms being scoped, grey literature (not peer reviewed) was not included in the review. Systematic reviews were included at the level of abstract and title screening, as well as key relevant studies, and primary studies were identified from their reference lists for inclusion at the full text screening stage. Expert consultation also provided a primary study. The publication date was restricted to 2002 (1st January)–2022 (24th June) at full text screening, because the most widely used definitions of spirituality internationally date from 2001 (de Brito Sena et al., 2021). Papers were limited to the English language.

### Study Selection

**Fig. 1:**
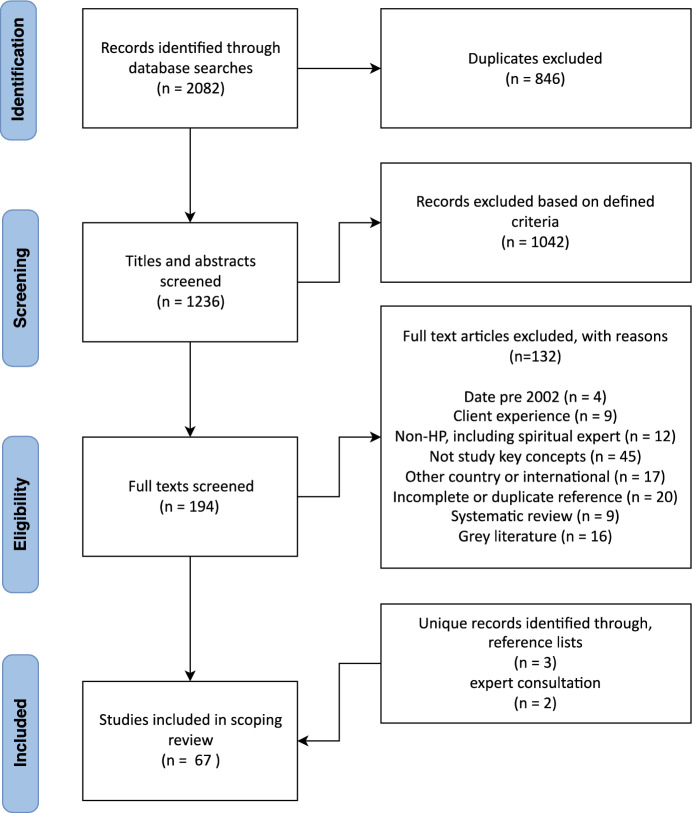
PRISMA-ScR flow diagram

The search results were collated and uploaded into Endnote X9 (EndNote, 2019) and transferred to Covidence (Veritas Health Innovation, 2014), a systematic review web application to manage the independent review process. A pilot screening of 100 randomly selected articles was performed between two researchers (HS, LM) to further refine inclusion criteria. All articles were then screened via title and abstract by HS and LM. Remaining articles were screened in full text by HS and a second reviewer (LM, CC, or JR). Any discrepancies that arose between the reviewers were decided by consensus. Figure 1 shows the full results of the search presented in the Preferred Reporting Items for Systematic Reviews and Meta-analyses for scoping reviews (PRISMA-ScR). Sixty-seven studies were included in the scoping review.

### Data Extraction and Analysis

Using an adaptation of the JBI template (Peters et al., 2020), data was extracted from included studies by HS, and was then reviewed by LM. Data extracted are summarised in Online Appendix II and included citation details, study objectives, context, profession, specialty, definitions related to key spirituality concepts, asking and responding actions, facilitators and barriers. Data extraction was an iterative process, as per the JBI recommendations (Peters et al., 2020). Data were then further analysed with NVivo Qualitative Data Analysis Software (QSR International, 2021). One reviewer (HS) undertook a qualitative synthesis of the articles, and developed a presentation of the results into themes (Braun & Clarke, 2021), which was verified at each stage by a second reviewer (LM). Face validity of the themes and conceptual model was then established by all authors.

Any studies related to HP practice, HP perspective and any training needs, were included in the scoping review. It was not always possible to separate studies of current from intended future practices (Best et al., 2019; Cooper et al., 2022; Jones et al., 2020; Keall et al., 2014a, 2014b; Kelly et al., 2008; Rombola, 2019), so these were considered together. Additionally, there were some topic reviews and theory development articles that were related to the integration of spirituality into practice (e.g. D’Souza, 2007; D'Souza & George, 2006; Hassed, 2008; Tse et al., 2005). However, it is unknown if recommendations from these reviews are being evaluated in practice. All the studies were included to provide a comprehensive overview of spirituality in Australian HP practice, which includes spirituality education that advocates practices and therefore is assumed to impact practice.

## Results

**Fig. 2:**
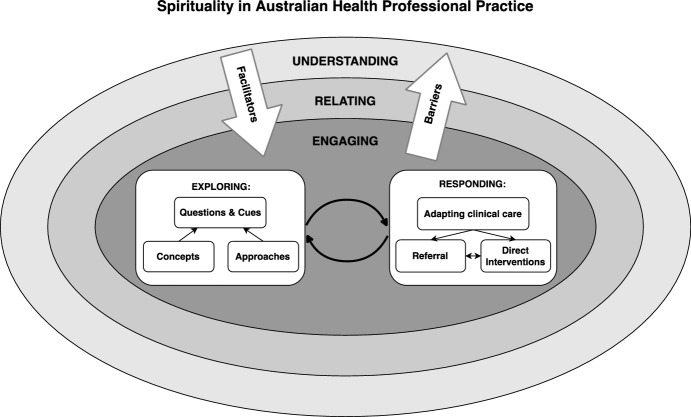
Spirituality in Australian HP practice

Sixty-seven articles were included in the review, see Fig. 1. Details of these results are included in Online Appendix II. Overall, most published studies were qualitative (n = 45); followed by quantitative (n = 12) and mixed methods (n = 10). Within the qualitative methods employed, semi-structured interviews made up over half of all studies (n = 22). Topic reviews (n = 10), theory development (n = 9) and education course summaries (n = 3) were a group of qualitative articles that collectively focused on spirituality education. Within the twelve quantitative studies, researcher-developed surveys were the dominant approach (n = 8); followed by adapted versions of the RNC Spirituality Survey (McSherry & Jamieson, 2011) (n = 2); the combined Spirituality and Spiritual Care Rating Scale (SSCRS) (McSherry et al., 2002) and the Spirituality Care Competency Scale (SCCS) (van Leeuwen et al., 2009) (n = 1); and the combined Spiritual Perspective Scale (SPS) (Reed, 1987) and Spiritual Care Practice Questionnaire (SCPQ) (Vance, 2001) (n = 1). To present the results of this study in a qualitative synthesis, a conceptual model was developed using thematic analysis. The overarching themes identified to answer the initial study questions were: ‘understanding’; ‘relating’; ‘engaging’ (‘exploring’ and ‘responding’); ‘facilitators and barriers’ to spirituality in Australian HP practice. The way these themes interact with each other are represented in the conceptual model Fig. 2.

‘Understanding’ of spirituality and its related concepts encompasses the whole model. It represents definitions, concepts of spirituality and the HP authors behind this ‘understanding’. This ‘understanding’ underpins all HPs interactions with a client’s spirituality. Moving from ‘understanding’, HPs talked about employing ‘relating’ skills, which were the relational and often non-verbal skills described to support client spirituality. Next, ‘engaging’ addresses the active and verbal addressing of a client’s spirituality by HPs. It covers the subthemes ‘exploring’ and ‘responding’. ‘Exploring’ presents the range of questions and cues interpretation (and the concepts and approaches underlying these questions) that HPs use to ask about a client’s spirituality. Three distinct HP ‘responding’ actions were identified. ‘Facilitators’ and ‘barriers’ describe the factors that influence understanding, relating to and engaging with a client’s spirituality.

### Theme One: Understanding of Spirituality and Related Concepts

There has been a rise in the HP perspective on spirituality in healthcare literature in Australia over the last twenty years, with thirty-seven articles in the last decade. Most articles originated from NSW (n = 23) and Victoria (n = 14), and from major cities (n = 39). Physical contexts included hospital (n = 19), university (n = 16), community settings (n = 12), mixed physical contexts (n = 13) and not stated/other (n = 7). Only two articles were focused on rural and remote areas; one article originated from WA, NT, and ACT, and none from Tasmania. The top three professions writing about spirituality in Australia were nursing (n = 21), medicine (n = 14) and social work (n = 13). There were two HPs identified as Aboriginal authors, and Aboriginal Health Workers were included as part of one multidisciplinary study. The most highly represented specialty areas were palliative care (n = 13), palliative care-related (n = 6) and Aboriginal health (n = 6), with half of the Aboriginal Health articles concerning palliative care. There were ten multidisciplinary participant studies. Most contexts were secular (n = 39) or not stated (n = 22), with a minority faith-based (n = 2) or mixed (n = 4).

### Spirituality

Forty-seven articles provided some type of summary definition of spirituality. The most popular definition of spirituality (n = 5) was the international palliative care consensus definition: spirituality is “the aspect of humanity that refers to the way individuals seek and express meaning and purpose and the way they experience their connectedness to the moment, to self, to others, to nature, and to the significant or sacred” (Puchalski et al., 2009, p. 887). In most articles (n = 25), HP authors provided a completely original definition, or one that was created from multiple other authors. All five Aboriginal health specialty articles provided unique definitions of spirituality that described its interrelatedness, for example, “the Aboriginal perspective incorporates a whole-of-life outlook which not only focuses on the social, emotional, spiritual and cultural well-being of the individual, but also of the entire community” (O'Brien et al., 2013, p. 5).

Within the forty-six Australian definitions used, concepts were divided into the subthemes identified by de Brito Sena et al. (2021): ‘connection’ (how spirituality is engaged), ‘core concepts’ (what spirituality is) and ‘well-being’ (effects of spirituality). Spirituality was most frequently defined using the ‘well-being’ concept, particularly as meaning (n = 22), and purpose in life (n = 18). Spirituality was also frequently described as ‘connection’ (n = 14). The ‘core concepts’ of spirituality were described as connection to community (n = 11), self (n = 9), nature/land/country (n = 9), divine/God/higher power (n = 7), significant or sacred (n = 6), Transcendent/spiritual world (n = 5), and the moment (n = 5). Additional descriptors included that spirituality was ‘part of a holistic health concept’ (n = 15), ‘part of being human’ (n = 10) and ‘humanity’s existential journey/pursuit/search’ (n = 8).

### Spiritual Care

‘Spiritual care’ had fewer stated definitions (n = 14), and these were predominantly authored from within nursing (n = 8). There was no overlap within the definitions of ‘spiritual care’ between authors, with each author presenting a distinctive vision. Definitions included: spiritual care “recognises and responds to the needs of the human spirit when faced with trauma, ill health or sadness…” (National Health Service Scotland, 2009, p. 6), it is “person-centred care” (NHS Education for Scotland, 2013; Spiritual Care Australia, 2013), and it is part of “holistic care” (Cooper & Chang, 2016, 2022).

### Religion

There were twenty-five articles that provided definitions for religion. Definitions were divided into three categories; (i) those that defined religion as a concept (n = 10); (ii) those that defined religion, often warily, in relation to other concepts such as spirituality (n = 11) or culture (n = 1); and (iii) those that defined religion as misunderstood (n = 3). As with spirituality definitions, there was little overlap between religion definitions. The most popular definition for religion, simply because it was used by two different authors, was: religion is “an institutionalised (i.e. systematic) pattern of values, beliefs, symbols, behaviours, and experiences that are oriented toward spiritual concerns, shared by a community, and transmitted over time in traditions” (Canda & Furman, 1999, p. 37; 2009, p. 59).

### Theme Two: Relating to a Client’s Spirituality

There were thirty-eight article references regarding ‘relating’, which encompassed the relational and non-verbal communication skills used by HPs that supported a client’s spirituality (National Health Service Scotland, 2009). Relating skills were mentioned by articles both as precursor to further ‘engaging’ with client spirituality (Keall et al., 2013; Pratt, 2007), and as an intervention in and of itself (Cooper & Chang, 2022; Keall et al., 2014a, 2014b; Ormsby & Harrington, 2003). These skills were most frequently described within the nursing literature. Relating skills were most often organised under the article title concept ‘spiritual care’ (n = 8) and secondly under the concepts ‘spirituality/spiritual/existential’ (n = 5).

‘Relating skills’ included ‘active listening’, ‘building rapport and relationship’, and ‘holding a client’s hand’. Active listening (n = 19) frequently included “listening to a patient’s life story” (Cooper & Chang, 2022) and physical “presence” (Lo, 2003) to convey “compassion” (Jones et al., 2020). Building rapport and relationship (n = 13) was described as not just going “in there cold” (Cooper & Chang, 2022), but taking the time to “get to know” a client (Estacio et al., 2018). Finally, holding a client’s hand (n = 6), “so they know they’re not alone” (Ormsby et al., 2017), was described solely in the nursing literature.

### Theme Three: Engaging with a Client’s Spirituality

‘Engaging’ addresses the active and verbal addressing of client spirituality by HPs and encompasses the two subthemes ‘exploring’ and ‘responding’.

#### Subtheme: Exploring

‘Exploring’ presents the range of direct questions and cue-following approaches used, or intended for use, to ask a client about their spirituality. There were fifty-two article references concerning this, with some articles referencing two–three different approaches (e.g. Best et al., 2019; Schreiber et al., 2022). The data was then organised regarding the total range of approaches (proactive to reactive) (see Table 1) and then the approaches’ conceptualisation of spirituality.

**Table 1:** Approaches to exploring a client’s spirituality

	Approaches to exploring a client’s spirituality	Number of Articles (N)
	**Proactive approaches**	
	***Spirituality-specific asking***	
	Spirituality assessment	2
	Spiritual history	5
	Spirituality models guiding clinical practice	4
	***Integrated asking***	
	Formal: comprehensive assessment (e.g. assessments, screeners, guidelines, clinical resources)	17
	Informal: general questions (within a healthcare interaction)	12
	**Reactive approaches**	
	Respond to client initiative or cues	11
	Respond after referral from another colleague or documentation	1

(N) represents the number of articles where that concept reference was found, with some individual articles containing multiple references

Firstly, approaches to asking were organised on a spectrum (see Table 1) from most pro-active in spirituality-specific asking (n = 11); to integrated asking within HP roles in formal and informal ways (n = 29); to only being undertaken reactively, after clients initiated the discussion (n = 12). Methods were categorised according to information from the article, however not all articles gave specific references, for instance the ‘spirituality assessment’ used in both articles were not named. The ‘spirituality-specific asking’ approaches were deemed the most proactive because, while many are recommended for use within a comprehensive healthcare assessment, they target spirituality and were lengthier in general. For example, the American College of Physicians’ spiritual history tool (Lo et al., 1999, p. 746) was the most referenced spiritual history tool (n = 3) and uses four questions: “(i) ‘Is faith (religion, spirituality) important to you?’, (ii) ‘Has faith been important to you at other times in your life?’, (iii) ‘Do you have someone to talk to about religious matters?’, (iv) ‘Would you like to explore religious or spiritual matters with someone?’”. In contrast, the formal approach of comprehensive assessment often either implied or quoted one-two questions; e.g. “(i) Does the patient have concerns about spiritual or existential issues?, (ii) Is the caregiver or family experiencing physical, practical, spiritual, existential, or psychological problems that are interfering with their well-being or functioning?” (Waller et al., 2013, p. 918). Informal approaches used general questions within a healthcare interaction, such as, “How are you making sense of what’s happening?” (Jones et al., 2020, p.6). Reactive approaches were those where the HP relied upon cues or initiative from the client, or referral, before asking further questions about spirituality.

Secondly, spirituality conceptual approaches were analysed on their wording for two general themes. Firstly, whether spirituality was described as ‘spiritual need/issues’ or ‘spirituality/inclusive of strengths’; then secondly whether spirituality was addressed solely to the individual, or to the individual within his/her social context. The ‘informal: general questions’ and ‘formal: comprehensive assessment’ were conceptually analysed and compared as they both provided the most detail in their approach. It became apparent that way spirituality was conceptualised to Australian clients changed depending on the mode of asking.

For Australian HPs, when using ‘informal: general questions’, ‘spirituality/inclusive of strengths’ was the dominant concept (n = 10). Whereas in ‘formal: comprehensive assessment’, ‘spiritual need/issues’ were the dominant concept (n = 10). Additionally, when using ‘informal: general questions’, spirituality was more frequently conceptualised as individual (n = 8); whereas in ‘formal: comprehensive assessment’, the individual was more frequently addressed in their family and community context (n = 10).

#### Subtheme: Responding

There were fifty article references regarding ‘responding’ to a client’s spirituality. After exploring a client’s spirituality, ‘spiritual beliefs and practices’ may have been identified by HPs, and these were often discussed by articles together alongside ‘religious and cultural beliefs and practices’. There were three general HP responding actions that could be identified in the literature: (i) HP adapted their own clinical care to accommodate client beliefs and practices (n = 19); (ii) HP referred to spiritual expert when client spiritual need was beyond the skill or comfort level of the HP (n = 13); and (iii) HP offered or discussed direct spiritual interventions with client (n = 18) (see Table 2).

**Table 2:** Responding to a client’s spirituality

Responding to a client’s spirituality	Number of articles (N)
**(i) Adapting clinical care to client spiritual, religious and/or cultural beliefs and practices:**	
General adapting care	9
Environmental adaptations (access to nature; prayer rooms or quiet spaces; family-gathering spaces; religious rituals)	3
Task adaptations (dietary needs; medication needs; modesty, pain, and hygiene differences; flexible clinical practice)	6
Post death care of the client’s body	1
**(ii) Referral to spiritual expert**:	
Chaplain, spiritual care practitioner or community spiritual leader	13
**(iii) Offer or discuss direct spiritual interventions:**	
**Health professional programs with a spiritual focus**	5
Spirituality in Mental Health (n = 1), Outlook Palliative Care Preparation and Life Completion Intervention (n = 2), Music or art therapy (n = 2)	
**Spiritual modalities**	13
Herbs, alternative therapies (n = 1), Meditation, mindfulness (n = 2), Psychic, astrology, spiritual healing (n = 1), Religion-accommodative psychology approaches (n = 1), Yoga, tai chi or reiki (n = 2), Prayer (n = 6)	

(N) represents the number of articles where that concept reference was found, with some individual articles containing multiple references

Whilst some articles were clear that in-depth spiritual issues should be handled by spiritual experts such as chaplains (D'Souza, 2007; D'Souza & George, 2006; Jantos & Kiat, 2007; Passmore, 2003; Peach et al., 2003), other articles made clear that Australian HPs were undertaking or discussing either HP designed spiritual interventions or spiritual modalities with clients (Jones et al., 2020; Keall et al., 2014a, 2014b; Lo, 2003; Lynn & Mensinga, 2015; Rice & McAuliffe, 2009).

### Theme Four: Facilitators and Barriers

Factors relating to facilitators and barriers for understanding, relating to, or engaging with client spirituality are presented in Table 3. Factors were roughly organised into human (HP or client related factors) or environmental (conceptual, policy and guideline, knowledge and training, or workplace context factors). Key facilitators of spirituality included HP training in spirituality (n = 36), a holistic care approach (n = 26), cultural and religious diversity approach (n = 20) and HP self-reflection on spirituality (n = 18). Key barriers reported by articles were a lack of spirituality and related concepts training (n = 19), lack of time (n = 17), difficult to define spirituality concepts (n = 15), and the physical focus of care (n = 12).

**Table 3:** Summary of facilitators and barriers

	Human	Environmental
**Facilitators**	‘Part of my role’ (n = 8)	***Conceptual Factors***
	Client spiritual need, crisis (n = 5)	Holistic care approach (n = 26)
	Clinically experienced HP (n = 4)	Cultural and religious diversity approach (n = 20)
	Spiritual or religious HP (n = 4)	Person-centred care approach (n = 11)
	Spiritual or religious client (n = 3)	Biopsychosocial-spiritual model (n = 5)
	Family involved with client (n = 3)	***Policy and Guideline Factors***
	Close relationship with client (n = 3)	International documents, e.g., WHO, UN, etc. (n = 13)
	Shared cultural background (n = 2)	Australian documents, e.g., Guidelines, etc. (n = 5)
		***Knowledge or Training Factors***
		Spirituality and related concepts training (n = 36)
		HP self-reflection on spirituality (n = 18)
		***Workplace Context Factors***
		Spirituality in comprehensive assessment (n = 9)
		Spaces that allow privacy or family gatherings (n = 4)
		Faith-based workplace (n = 3)
		Spirituality documented in client notes (n = 2)
		Spiritual expert availability (n = 2)
**Barriers**	HP feels uncomfortable, fearful, challenged (n = 11)	***Conceptual Factors***
	Cultural or language difference (n = 9)	Difficult to define spirituality concepts (n = 15)
	‘Not part of my role’ (n = 9)	Taboo or ignored concepts in society (n = 3)
	Socially disadvantaged client (n = 5)	***Australian Policy and Guideline Factors***
	New Graduate or < 5 years’ work (n = 4)	Only culture addressed (n = 4)
	Religious or spiritual difference (n = 3)	Spirituality only for indigenous Australians (n = 4)
	Lack of spiritual expert referral (n = 2)	Spirituality for some practice areas only (n = 3)
		Spirituality not mentioned (n = 2)
		***Lack of Knowledge or Training Factors***
		Spirituality and related concepts training (n = 19)
		Spirituality research (n = 8)
		Australian content (n = 3)
		***Workplace Context Factors***
		Lack of time (n = 17)
		Physical focus of care (n = 12)
		Lack of spirituality in holistic assessment tool (n = 7)
		Lack of spaces for privacy or family (n = 6)
		Lack of spirituality documented in client notes (n = 4)
		Covid-19 restrictions (n = 2)

(N) represents the number of articles where that concept reference was found, with some individual articles containing multiple references. ‘Spirituality and related concepts’ may include spirituality, spiritual care, religion, culture, and existential, as these concepts were often discussed together in articles

## Discussion

This study initially set out to answer how Australian HPs define key spirituality concepts; how they ask about and respond to their clients’ spirituality; and what the facilitators and barriers to addressing client spirituality are. The three key findings from this study were that ‘meaning’ was the most frequent concept in Australian HP spirituality definitions, and that ‘holistic care’ and ‘cultural and religious diversity’ were the most repeated organising concepts. Secondly, both relational (relating) skills and directly engaging (using questions and following cues, and then responding) were ways that client spirituality was addressed by HPs. Finally, over half of all articles reported professional training in spirituality as a facilitator, whereas lack of time was the most common barrier.

The high frequency of completely original and combined ‘spirituality’ definitions speaks to the general confusion and lack of consensus around this topic. ‘Spiritual care’ was not widely employed by this group of HPs, and there was no overlap of definitions between authors. In Australia, the language of ‘spiritual care’ may be confusing for spiritual generalist HPs to employ as it may be a term associated with spiritual care experts, particularly given that the peak body for chaplaincy and spiritual care practitioners is called ‘Spiritual Care Australia’. ‘Religion’ was also most frequently defined defensively, against other concepts or as misunderstood. For many, these topics can be taboo. An evidenced based approach to spirituality and its related concepts in Australia is essential, and this will include having a common vocabulary.

The most frequent concepts found in the Australian definitions of ‘spirituality’, found in nearly one third of definitions, were the well-being concepts of ‘meaning’ and ‘purpose in life’. In international spirituality definition concepts, ‘meaning/purpose’ is also significant, being found in half of all definitions (de Brito Sena et al., 2021). This is significant, as this defines spirituality both in Australia and internationally as representing humanity’s existential search for ultimate answers (Carey & Mathisen, 2018). Meaning in life is relevant to both the religious and non-religious and can become of pressing relevance to clients who are faced with their own suffering and mortality (Michael et al., 2020). However, Australian HPs also describe spirituality with a slightly different emphasis to their international counterparts. In ‘core concept’ descriptors, internationally ‘Divine/God/higher power’ and ‘Transcendence’ appeared more frequently (de Brito Sena et al., 2021), whereas in Australia, ‘community’, ‘self’ and ‘nature’ were marginally more frequent. This would appear to match Australia’s secularising trend over recent decades (Best et al., 2022a, 2022b).

For HPs in Australia, ‘holistic (whole person) care’, and ‘cultural and religious diversity’, were the dominant organising concepts for Australian HPs to integrate spirituality into practice. Internationally, a holistic care approach is a flexible vision of how the spiritual could be integrated with body and mind in healthcare (Southard, 2020). This holistic focus was illustrated in the connection concepts used within Australian definitions of spirituality, and in all five Aboriginal definitions. Here, “spirituality” was presented as interweaving the “whole-of-life” (O’Brien et al., 2013, p.5; Pratt, 2007, p. S54), echoing views reported by our Aboriginal communities (Grieves, 2009; Smith et al., 2021). Additionally, in this study, responses to a client’s spirituality were often discussed alongside religious and cultural beliefs and practices. This mirrors the impact of Australia’s steady growth in immigration and religious diversity (Australian Bureau of Statistics, 2022b), and may also be discussed in the literature under the term ‘cultural safety’ (Yeheskel & Rawal, 2019).

There are many voices missing from this discussion, and of particular importance is that the Aboriginal Health Worker perspective on spirituality in Australian healthcare is not well represented in the existing literature. There was also a lack of uniquely allied health authored articles, and this paucity of allied health research has also been noted internationally (Carey & Mathisen, 2018). Spirituality was frequently defined as part of being human, yet it was often discussed in Australia as having limited application. Specialties that dealt with imminent death were at the forefront of this spirituality discussion, and so spirituality can be seen as only relevant to the dying. Social work authors critiqued the limited scope of spirituality, highlighting its reduction to ‘culture’ (Gardner et al., 2020), being presented as only relevant to one cultural group (in particular, only to Aboriginal and Torres Strait Islanders) or as only for people in crisis situations (Crisp & Dinham, 2019). Spirituality has not been a concept applied equally to all people across all areas of Australian healthcare.

‘Relating skills’ were frequently described as non-verbal interactions. On occasions, these were the intervention. These skills were most frequently described within the nursing literature under the concept ‘spiritual care’. However, it should be noted that while these relating skills overlap elsewhere in the literature with descriptions found within concepts such as “attunement” (Kroier et al., 2020, p. 2), “compassionate care” (Puchalski et al., 2013, p. 58), and “therapeutic use of self” (Taylor, 2020, p. 12). Further research and conceptual consensus on relating skills terminology across health professions would be beneficial.

The reasons for choosing ‘relating skills’ as a discrete responding action were not specifically articulated in these Australian articles, however some have suggested that it may be based on a HPs “personal and professional expertise and training” (McColl, 2022, p. 743). These sensitive skills have been described as the “most basic way” to connect with clients on a “spiritual level” (McColl, 2022, p. 743). Nursing literature led the way, and internationally nurses are also at the front of describing, organising and creating training around these skills (van Leeuwen et al., 2021). Further research from other professions would be beneficial. For example, there were few articles led by psychology, none of which directly discussed these relating skills, and yet their professional contribution to this discussion would be invaluable.

The most frequently reported ‘exploring’ approach was to use a formal comprehensive assessment, including assessments, screeners, guidelines, or clinical resources. This is a practice not reported in a recent scoping review on current guidelines (Spiritual Health Association & Spiritual Care in Aotearoa New Zealand, 2022), yet it appears to be a format appropriate to the roles of spiritual generalist HPs. While internationally spiritual history appears to be a preferred format (Puchalski, 2021), it was interesting to note that popular tools such as the FICA (Puchalski, 2021) and HOPE (Anandarajah and Hight, 2001) were not mentioned in these Australian articles, and were unfamiliar to Australian HP and spiritual expert participants in another recent study (Jones et al., 2021). It is also significant that many articles reported both proactive and reactive approaches in different practice settings. Studies addressing education indicated that HP participants were exposed to ways of extending their practice, beyond their current approach. This may represent both a flexible approach to exploring client spirituality according to individual needs (Best et al., 2019; Pham et al., 2022), and a continued need for HP training and support (Morris et al., 2014).

This study investigated how spirituality is conceptualised to clients in Australia and found a mixed presentation. A similar mixed presentation is noted between the World Health Organisation’s International Classification of Functioning, Disability and Health (Australian Institute of Health & Welfare, 2002) and it’s Quality of Life Questionnaire (World Health Organisation, 1997), where spirituality is approached under two different conceptual headings; personal beliefs (WHOQOL-Bref) and as a social environment (ICF). To avoid a western individualised conception of spirituality (Fijal & Beagan, 2019), a recognition of the individual within their social networks is recommended. Spirituality was also conceptualised both as ‘spiritual need/issues’ or ‘spirituality/inclusive of strengths’. Spiritual needs often arise close to death (Mesquita et al., 2017), and the dominant deficits model focus of healthcare also lends itself to a needs-based focus (Kennedy et al., 2022; Swartz, 2017). Alternatively, a balanced vision of spirituality that is inclusive of strength aligns with areas of Australian healthcare outside of imminent death and crisis (Victorian Advocacy League for Individuals with Disability Inc., N.D.), and with the Aboriginal authored definitions of spirituality.

Responding to a client’s spirituality with specific environmental and/or task adaptations was under-reported and is an area for further research. There was split opinion about whether Australian HPs were the right people to be undertaking direct spiritual interventions with clients. However, because of the limited availability of chaplaincy in some health settings (e.g. in the community) or due to COVID-19 limitations on chaplaincy (Tan et al., 2021) some HPs may be more inclined to directly respond to a client’s spirituality. All the HP responding actions, although often described as ‘spiritual’, often also had a religious and/or cultural element (e.g., many of the ‘spiritual modalities’ being discussed with clients originated within religions). Given the growing popularity of eastern-origin spiritual modalities such as mindfulness and yoga (Sun et al., 2021), the ancient and enduring practice of prayer (Bentzen, 2021; Narayanasamy & Narayanasamy, 2008), some authors have undertaken to offer HPs ethical guidelines for when it is appropriate to offer direct spiritual interventions (McColl, 2022). That discussion is beyond the scope of this paper; however, it is important to note that direct spiritual interventions by HPs has both ethical and training implications.

The most prevalent barriers to addressing client spirituality for Australian HPs were lack of time and training; and HP feeling uncomfortable, fearful or challenged; which were consistent with dominant barriers found internationally (Koenig, 2015). The most frequently reported barriers to addressing a client’s spirituality was a ‘lack of time’, and this was followed by the ‘physical focus of care’. This was a concern heightened in recent articles commenting on covid-19 restrictions, where religious, cultural and family supports were seen as “non-essential” (Boyle et al., 2022, p. 3). There is a need for further research into the “time” and “place” (environment and resources) needed for client spirituality to practically supported in healthcare settings (Spiritual Health Association & Spiritual Care in Aotearoa New Zealand, 2022, p. 20).

The highest reported facilitator for HPs was access to spirituality and related concepts training, followed by HP self-reflection, and this has been reported internationally (Harrad et al., 2019; Pham et al., 2022). Despite the frequently articulated call for training, there were few postgraduate and undergraduate training opportunities described in the Australian literature, and this is an area for further growth. Australian HPs more frequently relied on international health documents and some authors noted the inconsistencies in local policies and guidelines. This is a problem observed in other countries (Whitehead et al., 2021), and would benefit from more development.

## Limitations of this Scoping Review

The intention of the review was an Australian focus which entails that this study does not take into full account the international situation. Studies written in languages other than English were excluded, however given that the population focus was Australia, this exclusion was deemed to be appropriate. All authors contributing to this article are occupational therapists, which impacts our interpretation of the data and subsequent theme development. It is acknowledged that, while data extraction and analysis was verified at each stage by at least one other reviewer, the initial process was undertaken by one reviewer, and this may limit reflexivity. This discussion of spirituality represents a particular window of time in Australian healthcare literature. In the future, it is hoped that the specialties and professions who are missing from the conversation will broaden the discussion.

The representation here of spirituality is a modern conception, removed from its historically religious definition (Koenig, 2015). This study therefore acknowledges that not all religious people will identify with this definition of spirituality. This study also represents HP perspectives on integrating client spirituality, and it is acknowledged that client perspectives are not discussed here but can be found in other studies (Best et al., 2022b). According to scoping review methods (Khalil et al., 2021), a broad range of approaches was collected, both current and intended future practice, including specifically referenced tools and general approaches. This study does not include a critical appraisal of each article, including the number or percentage of HPs who undertook each method, or their effectiveness in a particular setting.

## Conclusion

This study mapped how Australian HPs are understanding, relating to and/or engaging with client spirituality, and the multitude of contributing facilitators and barriers. Key findings were that ‘meaning’ and ‘purpose in life’ were the most repeated concepts in Australian HP definitions of spirituality, emphasizing spirituality representing humanity’s existential search for ultimate answers. A third of articles identified ‘holistic care’ and ‘cultural and religious diversity’ as important organising concepts. The most frequently reported approach for Australian HPs in asking about client spirituality was using one or two questions within a comprehensive assessment. Nearly half of articles identified additional HP training in spirituality as a key facilitator. Further primary research is required to better understand how spirituality may be integrated into practice, particularly from areas of healthcare that are under-represented.

### Electronic supplementary material

Below is the link to the electronic supplementary material.

Supplementary file1 (DOCX 68 KB)

## Appendix l: Medline Search 24th June 2022

Database: Ovid MEDLINE(R) ALL < 1946 to June 24, 2022 > 

Search Strategy:

1. Audiologists/ or Audiologist*.mp. (1884)
2. Exercise Physiologist*.mp. (411)
3. Physical Therapists/ or Physiotherapist*.mp. (11,266)
4. australia/ or australian capital territory/ or new south wales/ or northern territory/ or queensland/ or south australia/ or tasmania/ or victoria/ or western australia/ (163,438)
5. australia*.mp. (207,700)
6. Podiatrist*.mp. (1001)
7. Psychologist*.mp. (17,840)
8. Speech Pathologist*.mp. (835)
9. Social Workers/ or Social worker*.mp. (11,641)
10. nurse*.mp. (390,719)
11. healthcare worker*.mp. (18,223)
12. healthcare professional*.mp. (32,321)
13. healthcare practitioner*.mp. (2271)
14. spiritual*.mp. or Spirituality/ (24,083)
15. Religion/ or religio*.mp. (69,223)
16. healthcare personnel*.mp. (2594)
17. health personnel*.mp. or Health Personnel/ (199,425)
18. therapist*.mp. (45,826)
19. chinese medical practitioner*.mp. (25)
20. chiropractor*.mp. (1943)
21. counsellor*.mp. (2417)
22. Counselors/ or counselor*.mp. (8295)
23. dentist*.mp. or Dentists/ (137,432)
24. dietician*.mp. (2127)
25. medical radiation practitioner*.mp. (10)
26. Orthoptist*.mp. (343)
27. Orthotist*.mp. (240)
28. Prosthetist*.mp. (434)
29. Optometrists/ or Optometrist*.mp. (2472)
30. paramedic*.mp. (9437)
31. Pharmacists/ or Pharmacist*.mp. (44,091)
32. Sexual Assault Worker*.mp. (0)
33. physician*.mp. (622,344)
34. doctor*.mp. (143,861)
35. welfare officer*.mp. (78)
36. Nursing Staff/ or nursing staff.mp. (78,731)
37. medical staff.mp. or Medical Staff/ (39,696)
38. medical practitioner*.mp. (6891)
39. remedial gymnast*.mp. (61)
40. social educator*.mp. (39)
41. 4 or 5 (224,408)
42. 14 or 15 (84,307)
43. health worker*.mp. (25,263)
44. health practitioner*.mp. (6193)
45. 1 or 2 or 3 or 6 or 7 or 8 or 9 or 10 or 11 or 12 or 13 or 16 or 17 or 18 or 19 or 20 or 21 or 22 or 23 or 24 or 25 or 26 or 27 or 28 or 29 or 30 or 31 or 32 or 33 or 34 or 35 or 36 or 37 or 38 or 39 or 40 or 43 or 44 (1,494,146)
46. 41 and 42 and 45 (301)
