# Supplementary material for: Spirituality in Australian Health Professional Practice: A Scoping Review and Qualitative Synthesis of Findings
Source: J Relig Health. 2023 Jun 12;62(4):2297–322. doi: 10.1007/s10943-023-01840-5 (PMC10258742; doi:10.1007/s10943-023-01840-5)
Supplement: Supplementary file 1 — Supplementary file1 (DOCX 68 KB) [file 10943_2023_1840_MOESM1_ESM.docx]

| Author | Lead Author Profession | Contexts | Methodology Objectives summary | | N = Specialty | | Summary points relative to review questions |
| --- | --- | --- | --- | --- | --- | --- | --- |
| Almansour et al, 2017 | Pharmacy | NSW; Major city (Sydney); secular; community | Qualitative –  Semi-structured interviews | To explore the current practices of Australian community pharmacists relating to Ramadan. | 21 | - | (D) Nil specific.  (A&R) Most pharmacists respond to client cues.  (F) most would like more training; Recommends proactive screening.  (B) lack time; feel like a GP will handle the issues. Community setting lacks privacy. |
| Bahrami, 2011 | Nursing | SA; Major city (Adelaide); secular; hospital | Qualitative - Semi-structured interviews | To explore in-depth meanings and aspects of QoL for cancer patients as expressed by their nurses. | 10 | Oncology | (D) The ‘spiritual’ is defined as a domain of QoL and should be addressed with patients. Spirituality encompasses religion.  (A&R) Nil Specific.  (F) Health crises can be a catalyst for client’s talking about spirituality. |
| Best et al, 2019 | Medicine | Australia-wide; regions not stated; affiliation not stated; hospital  and community | Qualitative – tool development – trial of Question Prompt List (QPL) | This study assessed the impact of a QPL on spirituality discussions in Palliative Care (PC). | 15 | Palliative Care | (D) Nil specific.  (A&R) 3 approaches: 1. half the time, doctors raise spirituality (informal Qs); 2. 38% raised by patient, 10% by family (responding to cues). 3. trial of patient initiated QLP (patient resource).  (F) doctors raising the topic; ‘peace’ as a topic; health crisis; first meeting; client lives with someone.  (B) missing client cues in raising spirituality. |
| Best et al, 2022 | Medicine | NSW; Major city (Sydney); secular; hospital | Qualitative - Education course | This paper describes the evaluation of the Interprofessional Spiritual Care Educational Curriculum (ISPEC)©. HP and spiritual expert participants. | 10 | - | (D) Spiritual care definition.  (A&R) General questions used for asking.  (F) client health crisis.  (B) barriers are lack of Australian training material including Aboriginal and secular relevance. |
| Bridge, 2014 | Medicine | WA; Major city (Perth); secular; university | Qualitative - Education course | Summary of the "Spirituality, Suffering and Healing" undergraduate medical student course. Curriculum summary. | 36 | Palliative  care | (D) Everyone is spiritual.  (A&R) Nil specific.  (F) Curriculum summary: interviews with terminal patients, key spirituality literature readings, daily reflective journal. |
| Boyle et al, 2022 | Not stated | Australia-wide; regions not stated; affiliation not stated; hospital | Quantitative – researcher developed survey | To explore Australian health care providers’ perspectives of the impact of COVID-19 on the provision of care following stillbirth or neonatal death. | 35 | Perinatal bereave-ment | (D) Nil specific.  (A&R) Asking within a holistic assessment or clinical guideline: Asking all parents whether they have any religious, cultural, or spiritual needs and facilitating requests where possible.  (B) Covid-19 restrictions significant, examples given. |
| Burgess et al, 2004 | Medicine | SA; Mixed regions; secular; community | Qualitative - Resource Development | A booklet for GPs to use to talk to patients about death and dying. | 22 | GP | (D) Nil specific.  (A&R) Spirituality and religion listed as part of the holistic pamphlet on death and dying; however, the exact questions not given. |
| Bush & Bruni, 2008 | Not stated | Vic; Major city (Melbourne); affiliation not stated; community | Qualitative - semi-structured interviews | To explore the meaning of the phenomenon of spiritual care, as described by palliative care professionals drawn from a number of health disciplines. | 8 | Palliative  care | (D) spiritual care meaning described as questionable.  (A&R) Nil Specific.  (F) HP self-reflection on own spirituality and relational skills like ‘being there’ for others.  (B) lack of training. |
| Carrington, 2013 | Social work | Vic; Regional city (Geelong); secular; university | Theory Development | This article presents an integrated spiritual practice framework (ISPF) which is a framework for integrating spirituality within social work. | 1 | - | (D) Spirituality described within holistic model of care.  (A&R) An intra-social spiritual practice framework (ISPF), which recommends discussing direct spiritual intervention. |
| Cioffi, 2004 | Nursing | NSW; Major city (Sydney); affiliation not stated; hospital | Qualitative - Semi-structured interviews | To describe expert midwives’ experiences in providing care to clients from culturally diverse backgrounds in a midwifery unit in an acute care hospital. The study only focused on Chinese and Islamic background clients. | 12 | Midwifery | (D) Nil Specific.  (A&R) Nil Specific.  (F) knowledge of religious and cultural practices, i.e.. Chinese mothers' Yin and Yang out of balance after birth; Islamic mothers' need for modesty during birth and cleanliness prior to praying.  (B) religious and cultural practices different to health practices, i.e.. Too many visitors at one time or late into the night because it is Ramadan. |
| Cooper & Chang, 2016 | Nursing | NSW; Major city (Sydney); faith-based; university | Qualitative - Semi-structured interviews | This study was the second phase of a larger project that aimed to explore the impact of a subject on spiritual care on the perceptions undergraduate nursing students have in being prepared to provide competent spiritual care. | 6 | - | (D) Spirituality difficult to define, and training given to undergraduates via the concept of ‘spiritual care’.  (A&R) Nil Specific  (F) Curriculum: client assessment (not specified), alleviate spiritual distress, promote spiritual well-being. Lecture form, over 13 weeks. Positive student evaluations reported. |
| Cooper et al, 2022 | Nursing | NSW; Major city (Sydney); secular; regulatory board | Qualitative - Semi-structured interviews | In the context of this study, critical discourse analysis enables a clearer understanding of how spirituality is understood by the participants to be represented in the current RN Standards for Practice. | 3 | - | (D) Spirituality and spiritual care definitions. Spiritual care linked to holistic care movement in nursing.  (A&R) Nil specific.  (F) person-centred practice, holistic care.  (B) spirituality difficult to measure and more philosophical in nature; spirituality negatively perceived by politicians and others as unprofessional; perceived as only relevant to Aboriginal Australians. |
| Cooper et al, 2021 | Nursing | NSW; Major city (Sydney); secular; hospital | Qualitative - Semi-structured interviews | To investigate how nurses’ construct their understanding of spirituality and spiritual care and uncover the dominant discourses that shape their understanding. | 20 | - | (D) Spirituality definition in depth.  (A&R) Relating skills of spiritual care listed.  (F) holistic care and cultural and religious diversity approach; training; faith-based workplace.  (B) difficult to define, secular workplace, lack of time and busyness. |
| Cooper & Chang, 2022 | Nursing | NSW; Major city (Sydney); faith-based; university | Mixed methods - Spiritual Care Education Subject | To investigate how nurses’ construct their understanding of spirituality and spiritual care and uncover the dominant discourses that shape their understanding. | 44 | - | (D) Spirituality and spiritual care definitions.  (A&R) Relating skills of spiritual care listed, referral to spiritual expert.  (F) Training. Curriculum: spirituality in holistic care, historical context, religion and culture, health, well-being, suffering and spiritual care, other faith traditions. |
| Crisp, 2011 | Social work | Vic; Regional city (Geelong); secular; university | Qualitative - theory development | This paper considers the implications for social work education in respect of acknowledging the religious dimension of the lives of many service users. | 1 | - | (D) Spirituality and religion definition.  (A&R) General questions used to broach spirituality.  (F) HP understanding the potential role of religion in their client’s lives.  (B) HP feel uncomfortable about religion. |
| Crisp & Dinham, 2019 | Social work | Vic; Regional city (Geelong); secular; university | Qualitative - curriculum analysis | This article analyses "religion" within regulations and standards that frame social work education and practice across a set of English-speaking countries, as well as the Global Standards for the Education and Training of the Social Work Profession. | 2 | - | (D) Brief religion definition.  (A&R) Nil specific.  (B) guidelines for practice only relate to specific cultures (Aboriginal people) and specific practice areas (family violence). Religion and belief may frequently be addressed using over- arching frameworks such as ‘anti-oppressive’ or ‘anti-discriminatory’ practice. |
| D'Souza & George, 2006 | Psychiatry | Vic; Major city (Melbourne); secular; university | Qualitative - theory development | The phenomenology of spirituality and its relevance to psychiatry is considered, the concept of the psychiatrist and the clinician as a healer visited, and the evidence for the need for spirituality and religiosity for patients examined. | 2 | Mental health | (D) Spirituality is humanity’s inner realm. Religion described both for what it is, and as misunderstood. (A&R) Lo et al.’s Spiritual History (holistic assessment).  (F) Referral to chaplaincy advised. Prayer and ethical advice.  (B) Western medicine has dichotomized the mind, body, and spirit. Lack of research, few training resources outside of palliative care and ethics. |
| D’Souza, 2007 | Psychiatry | Vic; Major city (Melbourne); secular; university | Qualitative - theory development | A summary of how to apply spirituality into medical practice. | 1 | Mental health | (D) Spirituality and religion definitions.  (A&R) Lo et al.’s Spiritual History (holistic assessment).  (F) Referral to chaplaincy advised. Prayer and ethical advice.  (B) Western medicine has dichotomized the mind, body, and spirit. Lack of training. |
| Daher et al, 2015 | Pharmacy | NSW; Major city (Sydney); secular; community | Qualitative - Semi-structured interviews | To explore the frequency and nature of cases where patient’s articulated religious/spiritual belief affect medicine use; pharmacist perspectives on handling these issues and their resource needs for future practice. | 21 | - | (D) Spirituality and religion definition.  (A&R) Most pharmacists respond to client cues.  (F) clear labelling of medications for Jewish and Islamic patients, training in religious issues impacting medications.  (B) Lack of pharmaceutical industry transparency about medication ingredients; lack of training. |
| Estacio et al, 2018 | Medicine | NSW; Major city (Sydney); affiliation notstated; comm/hosp/uni | Qualitative - Semi-structured interviews | To investigate palliative care physicians’ understanding of symptom meaning, and their experiences of the discussion of symptom meaning with patients. | 17 | Palliative  care | (D) Nil Specific  (A&R) follow patient cues, relational skills (build relationship).  (B) professionalism; HP uncomfortable; lack of time, privacy, training; language barriers; physical focus of care. |
| Gardner, 2020 | Social work | Vic; Major city (Melbourne); secular; university | Qualitative - theory development | A personal 20-year reflection on how social workers generally feel uncomfortable in including religion, or even more broadly spirituality, in how they practise, in the Australian context. | 1 | - | (D) Spirituality and religion definition.  (A&R) Nil specific.  (F) faith-based organisation, nursing, Australian Association of Social Workers (2014) code of ethics, student exploration of religion and Indigenous spirituality.  (B) religion/spirituality only discussed in relation to culture in social work curriculum. |
| Harrington, 2006 | Nursing | SA; Major city (Adelaide); secular; hospital | Mixed Methods | From the perspective of the nurse and other health care providers, what constitutes spiritual care giving? | 13 | Palliative  care | (D) Spirituality, spiritual care, and religion defined. (A&R) Relational skills described: active listening, building relationships, adapting care.  (F) openness to other worldviews.  (B) difficult to define terms. |
| Hassed, 2008 | Medicine | Vic; Major city (Melbourne); secular; university | Qualitative - topic review | Summary of the role of spirituality in medicine. | 1 | - | (D) Spirituality and religion defined. Religion an “organised community of faith”.  (A&R) Lo et al.’s spiritual history. Referral to chaplaincy encouraged as a response.  (F) training.  (B) HP and societal negative views of religion. |
| Hegarty et al, 2005 | Nursing | SA; Major city (Adelaide); secular; hospital | Mixed methods | To study discrepancies between nurses’ verbal interview reports and their documentation of end-of-life care in acute wards. | 40 | Palliative  care | (D) Spirituality within holistic care approach.  (A&R) Nil specific.  (F) HP self-reflection.  (B) lack of documentation; no formal tool that includes religious, spiritual, or cultural section; time/busyness. |
| Holden, 2012 | Social work | Vic; Regional city (Bendigo); mixed affiliation; unknown physical context | Qualitative - Semi-structured interviews | This paper discusses findings of six social workers working in a large Australian regional centre who implement spirituality in their practices. | 6 | - | (D) Spirituality and religion defined; religion described as a secondary response to spirituality.  (A&R) respond to client cues.  (F) HP self-reflection.  (B) lack of training. |
| Hudson et al, 2012 | Nursing | Vic; Major city (Melbourne); mixed; comm/hosp/uni | Mixed Methods - Guideline Development | To develop clinical practice guidelines for the psychosocial and bereavement support of family care- givers of palliative care patients. | 11 | Palliative  care | (D) Nil specific.  (A&R) part of formal holistic assessment. Specific tool/questions not named. Assessing for spiritual needs is part of the holistic assessment for needs in palliative care patients.  (F)(B) Nil specific |
| Jantos & Kiat, 2007 | Medicine | SA; Major city (Adelaide); secular; community | Qualitative - topic review | This article explores four possible mechanisms by which prayer may lead to improved health. | 2 | - | (D) Spirituality defined.  (A&R) Ask general questions. Responding: refer to chaplains.  (B) lack of research; difficult to study. Prayer: Patients’ requests for prayer need to be addressed in the context of the wishes of the individual, the beliefs of health professionals, and the practice guidelines of a given institution. |
| Jensen & Phillips, 2013 | Medicine | ACT; mixed regions; affiliation not stated; hosp & comm | Qualitative - Semi-structured interviews | This study explores the attitudes of Australian evangelical Christian doctors to healing, suffering and good practice. | 13 | - | (D) Nil specific.  (A&R) Questions in patient questionnaires or general questions (re: personal beliefs and community).  (F)(B) Nil specific. |
| Jessop & Phelan, 2022 | Medicine | SA; Major city (Adelaide); secular; hospital | Quantitative – researcher developed survey | To identify barriers in the provision of optimal palliative care to Aboriginal children with cancer. | 34 | - | (D) Spirituality located within a holistic view of life.  (A&R) Nil specific.  (F) Every HP participant wanted more spiritual and cultural training; more space; cultural insider/AHW; aboriginal spiritual healers.  (B) language, culture, time; lack of knowledge. |
| Johns et al, 2019 | Social work | QLD; Rural & remote; affiliation not stated; community | Qualitative - Semi-structured interviews | The two aims of this research were to investigate psychosocial care provision for palliative clients in rural communities and to identify barriers and facilitators of social work referrals. | 33 | Palliative care | (D) ‘Spiritual’ located within the bio-psycho-social-spiritual model and enveloped within the psychosocial concept.  (A&R) Nil specific.  (F) holistic care approach.  (B) pragmatics-focused assessment. Spirituality not clearly defined, appeared lost within ‘psychosocial’. |
| Jones et al, 2019 | Nursing | QLD; Major city (Brisbane); secular; hosp & uni | Qualitative - theory development | A philosophical discussion of spirituality within nursing practice. | 4 | - | (D) Spirituality defined.  (A&R) framework is person-centred care, covering physical, emotional, mental, and spiritual needs.  (F)(B) Nil specific. |
| Jones et al, 2020 | Social work | NSW; Major city (Sydney); not stated; hospital | Quantitative - (SSCRS, McSherry; SCCS, van Leeuwen et al.) | To evaluate a spiritual care training program for rehabilitation professionals. | 73 | Rehabil-itation | (D) Spirituality, spiritual care, and religion itself defined.  (A&R) general questions; asking in initial assessment; active listening.  (F) Training; self-reflection.  (B) spiritual expert referral; documentation in notes. |
| Jones et al, 2020 | Social work | NSW; Major city (Sydney); secular; hospital | Qualitative - Semi-structured interviews | This study explored the impact of a brief spiritual care training program upon the perceptions and self-reported practice of rehabilitation professionals working in traumatic injury. | 16 | Rehabil-itation | (D) Spirituality, spiritual care, and religion (in relation to spirituality) defined.  (A&R) general questions; adding question to assessment forms.  (F) seeing it as part of your role; clients want to talk about it; self-reflection.  (B) physical focus of care; ongoing need for training. |
| Jones et al, 2020 | Social work | NSW; Major city (Sydney); secular; hospital | Quantitative - (SSCRS, McSherry) | To increase understanding of the perceptions of spirituality held by rehabilitation health professionals from a broad range of clinical disciplines and rehabilitation services. | 125 | Rehabil-itation | (D) Spirituality, spiritual care, and religion (in relation to spirituality) defined.  (A&R) Multiple methods of asking reported: observing cues or from referral, minority use spiritual assessment.  (F) training; consulting with spiritual expert.  (B) lack of training; discomfort; not part of my role; risk of discrimination; workplace policy. |
| Kang, 2003 | Occupational therapy | QLD; Major city (Brisbane); secular; university | Qualitative - theory development | This paper aims to elucidate the foundational principles and constructs underlying a new practice framework the psychospiritual integration (PSI) frame of reference. | 1 | - | (D) Many definitions of spirituality compared; however, a definition is given that focuses on well-being and connection concepts. Religion is defined against spirituality.  (A&R) frame of reference. Facilitators: holism and humanism; prior research & concepts; OT models.  (B) little research into ‘spiritual occupations’; difficult concepts; ‘not my role’, lack of training. |
| Keall et al, 2014 | Nursing | NSW; mixed regions; affiliation not stated; hosp & comm | Qualitative - Semi-structured interviews | To explore Australian palliative care nurses’ perceptions of the facilitators, barriers and strategies to provision of spiritual/ existential care to their patients. | 20 | Palliative care | (D) Nil specific.  (A&R) general questions. Relational skills needed (building rapport, etc).  (F) referral to social work, pastoral care worker or psychologist; realistic expectations about your own role; physical environment; documentation; training. (B) lack of time; fear; lack of privacy; difference of beliefs; lack of documentation; lack of clinical skill. |
| Keall et al, 2013 | Nursing | NSW; Major city (Sydney); affiliation not stated; hosp & comm | Qualitative - mixed methods | To explore the feasibility of a nurse facilitating the Outlook (Preparation & Life Completion) intervention and its potential applicability for use in clinical practice. | 5 | Palliative care | (D) Spirituality (Puchalski, 2009) and religion (in relation to spirituality) defined.  (A&R) Relational skills described. This is a direct HP spiritual intervention.  (F) training & debriefing; involving family when appropriate.  (B) negative client past experiences; communicating clearly takes time. |
| Keall et al, 2014 | Nursing | NSW; mixed regions; affiliation not stated; hosp & comm | Qualitative- semi-structured interviews | We asked Australian palliative care nurses how they perceived spiritual/existential interventions (in particular, Outlook) and if they perceived it would be feasible for nurses to deliver. | 20 | Palliative care | (D) Nil specific.  (A&R) general questions used. Participants reported that in their settings chaplaincy, social work and volunteers were addressing spirituality; yet the study then posed that nurses should give direct spiritual intervention.  (F) training.  (B) lack of time; new patient. |
| Kelly et al, 2008 | Medicine | QLD; Major city (Brisbane; affiliation not stated; hosp & comm | Qualitative - Semi-structured interviews | This qualitative study investigated psychosocial issues faced by GPs in the management of patients receiving palliative care. | 15 | GP | (D) Nil specific.  (A&R) responding to client cues.  (B) not part of my role. |
| Kichenadasse et al, 2017 | Medicine | Australia-wide; regions not stated; affiliation not stated; hosp & comm | Quantitative - survey - (SSCRS, McSherry) | To explore the current practice, preparedness and education of Australian oncologists and oncology trainees on the provision of spiritual care for their patients with cancer. | 69 | Oncology | (D) Spirituality (Puchalski, 2009), spiritual care, religion (defined in relation to spirituality).  (A&R) Nil specific.  (B) not my role; lack of time; lack of training. |
| Lloyd, 2004 | Medicine | NSW; region not stated; affiliation not stated; physical context not stated | Qualitative - topic review | A summary of addressing spiritually with the person with dementia. | 1 | Aging and Dementia | (D) Spirituality and religion (separate to spirituality) defined (unusual).  (A&R) Relational skills: active listening.  (F) training. Education resource: Dreher 1987 - Hierarchy of the spiritual needs.  (B) Australians can be private about spirituality; current training resources don’t address spirituality. |
| Lo, 2013 | Nursing | Australia; region not stated; affiliation not stated; physical context not stated | Qualitative - topic review | Summary of prayer as an integral component of the spiritual life of mankind. | 1 | - | (D) Spirituality and spiritual caring (presumably within nursing) defined.  (A&R) Relational skills: presence, touch, active listening, ?self-reflection. Asking: documentation of religious beliefs.  (F) quiet environment and privacy.  (B) challenging aspect of care. No clear recommendations. |
| Lynn & Mensiga, 2015 | Social work | QLD; Regional city (Cairns); affiliation not stated; physical context not stated | Qualitative - narrative analysis | To explore the integration of mindfulness in social work, we collected written stories from social workers who participated in two workshops in regional Australia. | 17 | - | (D) Nil specific.  (A&R) Relational skills described: active listening, presence.  (F) HP self-reflection and own spiritual practices; awareness of research.  (B) lack of time; physical focus of care. |
| McGrath & Phillips, 2008 | Social work | NT; Rural & Remote (Across regions); secular; community | Qualitative - Semi-structured interviews | What palliative care services are provided and are they meeting the clients’ needs? | 41 | Palliative care | (D) Spirituality is connected to holistic view of life.  (A&R) Nil specific.  (F) shared culture with client (AHW); acceptance of client’s cultural practices.  (B) non-acceptance of cultural practices; cultural insider knowledge. |
| Mitchell & D’Amore, 2021 | Not stated | Australia-wide; regions not stated; secular; community | Quantitative - researcher developed survey | This study explored the views of experienced GPs and practice nurses (PNs) towards the older-person HA forms. | 39 | Aging and Dementia | (D) Nil Specific.  (A&R) Nil Specific.  (F) holistic assessment.  (B) not part of current older person’s assessment. |
| Morgan et al, 2019 | Occupational therapy | Australia-wide; regions not stated; affiliations not stated; physical contexts not stated | Quantitative – researcher developed survey | AH clinicians’ perspectives, understanding and experiences about palliative care. | 187 | Palliative care | (D) Nil Specific.  (A&R) formal holistic assessment.  (F) part of my role; comfortable with suffering; holistic approach.  (B) AH do not routinely address spirituality and suffering. |
| O’Brien et al, 2013 | Not stated | NSW; mixed regions; secular; hosp, comm, uni | Qualitative - topic review | This review discusses palliative care and end-of-life models of care for Aboriginal people in the Australian state New South Wales and considers Aboriginal palliative care needs. | 9 | Aboriginal palliative care | (D) The land is defined as spiritual.  (A&R) holistic model of care.  (F) ‘whole-of-life outlook.  (B) language and cultural barriers; physical environment that supports family gathering. |
| Ormsby & Harrington, 2003 | Nursing | SA; Major city (Adelaide); secular; hospital | Quantitative - researcher developed survey | Definitions and methods of spiritual care for military nurses. | 35 | Military | (D) Spirituality and religion (in relation to spirituality) defined.  (A&R) professional model – Moberg, 1979.  (F) Family and a sense of belonging.  (B) spirituality not clearly defined. |
| Ormsby et al, 2016 | Nursing | NSW; Major city (Sydney); secular; hospital | Qualitative - semi-structured interviews | To explore the experience of spirituality and spiritual care by military nurses on deployed operations. | 10 | Military | (D) Spirituality and spiritual care defined.  (A&R) Relational skills: listening, taking time, respect, holding their hand.  (B) lack of time; clients who are not spiritual or religious; socially disadvantaged clients. Crisis scenario. |
| Parish et al, 2006 | Nursing | SA; Major city (Adelaide); secular; hospital | Mixed Methods - retrospective analysis | This study aimed to analyse the end of life care received by patients in the acute wards of a busy teaching hospital. | 40 | Palliative care | (D) Nil Specific.  (A&R) Asking: Assessment dimensions of care: physical, psychological, social and spiritual aspects of care.  (B) physical focus of care; lack of documentation. |
| Passmore, 2003 | Psychology | QLD; Regional city (Toowoomba); secular; university | Qualitative - topic review | Summary arguing that consideration of religious issues is an important aspect of therapy. | 1 | - | (D) Spirituality and religion defined.  (A&R) holistic assessment.  (F) holistic care & cultural and religious diversity approach, self-reflection.  (B) stereotyped views of religion and religious people, “valueless approach”, Australian codes of ethics only mention culture, lack of training, lack of research. |
| Peach, 2003 | Medicine | Vic; Regional city (Ballarat); secular; university | Qualitative - topic review | Religion, spirituality and health: how should Australia’s medical professionals respond? | 1 | Public health | (D) Spirituality and religion defined.  (A&R) Nil Specific.  (F) education.  (B) limited Australian research; lack of time; HPs feel uncomfortable. |
| Pek & Grocke, 2016 | Music therapy | Australia-wide; mixed regions; secular; physical contexts not stated | Quantitative - researcher developed survey | To investigate the influence of religious background and spirituality among Registered Music Therapists (RMTs) and clinical practice in Australia. | 45 | - | (D) Spirituality defined.  (A&R) Nil Specific.  (F) “part of my role”; self-reflective practice; accepting of differences; being religious or spiritual HP.  (B) “not part of my role”. |
| Pigott et al, 2009 | Psychology | Vic; Major city (Melbourne); secular; hospital | Mixed Methods - screening tool development | The specific aims of the current study were to develop a supportive needs screening tool | 41 | Oncology | (D) Spirituality within holistic view of a person.  (A&R) formal holistic screening tool.  (F) mass screening led to 30% of clients being identified with spiritual needs.  (B) no subsequent spiritual expert referrals were made, even when needs identified. |
| Pratt, 2007 | Aboriginal mental health worker | QLD; Regional city (Cairns); secular; community | Qualitative - theory development | Reflections of an Indigenous counsellor: sharing the journey | 1 | Aboriginal Health | (D) Spirituality within holistic view of life – whole of life view.  (A&R) Relational skills. Asking: holistic framework that takes in the whole of the individual’s life – community, culture, spirituality.  (F) holistic care.  (B) political forces past and present (disadvantaged clients). |
| Ranse et al, 2016 | Nursing | Australia-wide; regions not stated; affiliations not stated; physical contexts not stated | Quantitative - researcher developed survey | To identify factors associated with critical care nurses’ engagement in end-of-life care practices. | 392 | Critical care | (D) Spirituality within a holistic model of care.  (A&R) Nil Specific.  (F) standardised documentation of holistic assessment; more clinical experience; more training. (B) less clinically experienced. |
| Redfern & Bennett, 2022 | Social work | QLD; major city (Brisbane); secular; university | Qualitative - theory development | An intercultural critical reflection model: a new model which integrates, for the first time, both Western and Aboriginal Peoples’ epistemologies in critical reflection. | 2 | Aboriginal health | (D) Spiritual part of a holistic concept.  (A&R) Nil Specific.  (F) self-reflection; openness to learning.  (B) education resources not Australian; culture is a difficult concept. |
| Rice & McAuliffe, 2009 | Social work | QLD; mixed regions; secular; community | Qualitative - topic review | Ethics of the Spirit: Comparing Ethical Views and Usages of Spiritually Influenced Interventions with members of the Australian Association of Social Workers. | 2 | - | (D) Spirituality and religion defined.  (A&R) Discussion of direct spiritual interventions mentioned in depth.  (F) spiritual or religious HP; self-reflection; faith-based workplace; client-initiated discussion with general spiritual issues.  (B) power imbalance; “value-free” approach; not addressed in undergrad. |
| Rombola, 2019 | Medicine | NSW; major city (Sydney); not stated; community | Quantitative - researcher developed survey | The experience of Australian doctors using spiritual history-taking skills in holistic medical consultations and evaluated the support of Australian doctors for education. | 147 | GP | (D) Spirituality defined.  (A&R) Asking: spiritual history.  (F) bio-psycho-social-spiritual model; WHO docs; recognising patients want to talk about spirituality; knowledge of other religions and cultures; self-reflection; communication training.  (B) research largely American; Australian guidelines only address culture; hard to define concepts; spirituality related to only certain care scenarios; lack of time; lack of skill and training. |
| Ronaldson et al, 2012 | Nursing | NSW; major city (Sydney); secular; hospital | Quantitative - survey - (SPS) (Reed 1987) (SCPQ) (Vance 2001). | Spirituality and spiritual caring: nurses' perspectives and practice in palliative and acute care environments | 92 | Palliative care; acute care | (D) spirituality (it’s historically religious definition) and spiritual care defined.  (A&R) Nil Specific.  (F) Professional guidelines inclusive of spirituality (International Council of Nurses 2003, Australian Nursing & Midwifery Council 2006, Black et al. 2008, Nursing & Midwifery Council 2010); more clinical experience; being spiritual or religious HP; self-reflective HP.  (B) lack of time; privacy; less experience; lack of self-reflection. |
| Ronaldson et al, 2017 | Nursing | NSW; major city (Sydney); secular; hospital | Qualitative – open-ended survey questions | To investigate spiritual caring by palliative care nurses and to describe their interventions. | 42 | Palliative care | (D) Spirituality and spiritual care defined.  (A&R) Relational skills: active listening. Asking: On admission, cultural, religious, and spiritual priorities are identified.  (F) HP self-reflection; referral to chaplaincy; more experienced.  (B) less experienced; disengaged. |
| Schreiber et al, 2022 | Nursing | NSW & SA; mixed regions; secular; community | Qualitative - Semi-structured interviews | To study views on spirituality, spiritual care experiences, and descriptions about any spiritual care provided were collected from eight practice nurses. | 8 | Practice nurse | (D) Spiritual care (Narayanasamy, 2010).  (A&R) Relational skills: active listening, adapting care. Facilitators: HP self-reflection, client family member present.  (B) lack of time, training; physical focus of care; lack of holistic assessments; spirituality not in nursing standards. |
| Smyth & Allen, 2011 | Not stated | Vic; Regional city (Sale); secular; hospital | Mixed methods | To explore and describe how nurses define spirituality and incorporate spiritual care into their clinical practice. | 16 | Palliative care; acute care | (D) Spirituality and religion (in relation to spirituality) defined.  (A&R) Relational skills. Asking: following patients cues.  (F) recognising religious and cultural expression; heightened spiritual needs in health crises.  (B): difficult to define terms; lack of privacy, time, training; physical focus of care. |
| Tse et al, 2005 | Occupational  therapy | QLD; major city (Brisbane); secular; university | Qualitative - topic review | Exploration of Australian and New Zealand indigenous people's spirituality and mental health | 2 | Aboriginal health | (D) Spirituality linked to culture and community, whole of life view.  (A&R) Theoretical model (OT); mental health assessment in OT.  (F) holistic care approach; spiritual and cultural awareness.  (B) power imbalance; lack of research; HP “not part of my role”. |
| Voss et al, 2021 | Not stated | Vic; Major city (Melbourne); affiliations not stated; hosp & comm | Qualitative - Semi-structured interviews | The aim of this study was to explore health practitioners’ perspectives and practices relating to end-of-life decision-making and planning for people with PIMD. | 7 | Palliative care; Disability | (D) Nil Specific.  (A&R) Relational skills: active listening; building relationship.  (F) person-centred approach; UN convention on the rights of persons with disabilities.  (B) physical focus of care; lack of choice and support. |
| Waller et al, 2013 | Psychology | NSW; major city (Sydney); secular; hospital | Mixed Methods - screening tool development | To explore the psychometric quality of a newly developed rapid screening measure to assess the supportive and palliative care needs of people with CHF. | 21 | Cardiac | (D) Nil Specific.  (A&R) Asking: formal holistic assessment - client and family are asked re: spiritual issues.  (F) “part of my role”; holistic screening tool availability.  (B) potentially difficult to assess. |
| Wilding, 2002 | Occupational therapy | NSW; Regional city (Albury); secular; university | Qualitative – topic review | This paper discusses definitions of spirituality, barriers to practice, and argues for spirituality to be included within OT practice. | 1 | - | (D) Urbanowski & Vargo OT definition of spirituality. Religion defined against spirituality.  (A&R) Nil Specific.  (F) holism; OT models of practice; previous research & theory.  (B) lack of conceptual clarity; lack of education; physical focus of work, lack of time. |
